# The Role of Movement Analysis in Diagnosing and Monitoring Neurodegenerative Conditions: Insights from Gait and Postural Control

**DOI:** 10.3390/brainsci9020034

**Published:** 2019-02-06

**Authors:** Christopher Buckley, Lisa Alcock, Ríona McArdle, Rana Zia Ur Rehman, Silvia Del Din, Claudia Mazzà, Alison J. Yarnall, Lynn Rochester

**Affiliations:** 1 Institute of Neuroscience/Institute for Ageing, Newcastle University, Newcastle Upon Tyne NE4 5PL, UK; christopher.buckley2@newcastle.ac.uk (C.B.); Lisa.Alcock@newcastle.ac.uk (L.A.); R.Mc-Ardle2@newcastle.ac.uk (R.M.); Rana.zia-ur-Rehman@newcastle.ac.uk (R.Z.U.R.); Silvia.Del-Din@newcastle.ac.uk (S.D.D.); alison.yarnall@newcastle.ac.uk (A.J.Y.); 2 Department of Mechanical Engineering, Sheffield University, Sheffield S1 3JD, UK; c.mazza@sheffield.ac.uk; 3 The Newcastle upon Tyne Hospitals NHS Foundation Trust, Newcastle Upon Tyne NE7 7DN, UK

**Keywords:** movement science, Parkinson’s disease, ataxia, dementia, machine learning, deep learning, risk prediction, disease phenotyping

## Abstract

Quantifying gait and postural control adds valuable information that aids in understanding neurological conditions where motor symptoms predominate and cause considerable functional impairment. Disease-specific clinical scales exist; however, they are often susceptible to subjectivity, and can lack sensitivity when identifying subtle gait and postural impairments in prodromal cohorts and longitudinally to document disease progression. Numerous devices are available to objectively quantify a range of measurement outcomes pertaining to gait and postural control; however, efforts are required to standardise and harmonise approaches that are specific to the neurological condition and clinical assessment. Tools are urgently needed that address a number of unmet needs in neurological practice. Namely, these include timely and accurate diagnosis; disease stratification; risk prediction; tracking disease progression; and decision making for intervention optimisation and maximising therapeutic response (such as medication selection, disease staging, and targeted support). Using some recent examples of research across a range of relevant neurological conditions—including Parkinson’s disease, ataxia, and dementia—we will illustrate evidence that supports progress against these unmet clinical needs. We summarise the novel ‘big data’ approaches that utilise data mining and machine learning techniques to improve disease classification and risk prediction, and conclude with recommendations for future direction.

## 1. Introduction

Quantifying movement through clinical observation is central to enhancing our understanding of neurological disorders. It informs diagnosis, disease severity, progression, and therapeutic response. Mobility deficits (particularly gait and postural control, which form the focus of this review) provide critical information relevant to the diagnostic process. The clinical assessment of gait and posture within an outpatient, inpatient, or rehabilitation setting typically takes the form of self-report, subjective scales, and observation. Validated gait and postural control rating scales and assessments are also commonly applied (e.g., the Tinetti Performance-Oriented Mobility Assessment [1], the Dynamic Gait Index [2], and the Berg Balance Scale [3,4]). Although helpful in terms of change over time and considered the gold standard in clinical settings, significant limitations exist, due to variation, subjectivity, inconsistency, and poor granularity. Moreover, as we move to an era where the focus is on earlier detection, different tools are needed with greater sensitivity to detect change than is currently offered. 

Traditional approaches to quantify gait and postural control have relied upon complex and expensive laboratory equipment and specialist expertise, which lack translation to the clinic. Developments in movement analysis devices such as reduced prices and improved automated software are now facilitating their applicability not only to clinics but also for continuous monitoring on a large scale within real-world settings. The opportunity for better protocol standardisation, the harmonisation of outcome measures, and assessments of large cohorts through multi-centre studies in this evolving field are a welcome consequence. Equally welcome is the opportunity for improved stratification for clinical trials of novel neuroprotective therapies and disease progression. In an era of personalised medicine and early detection of risk, subtle changes in movement consequent to neurodegenerative disease would also improve clinical management through timely and accurate diagnosis and tracking, disease stratification, risk prediction, and enhanced decision making for intervention optimisation and maximising therapeutic response (such as medication selection, disease staging, and targeted support). In turn, the improved information provided with the correct interpretation may improve independence, quality of life, and a reduction of fall risk for patients.

Throughout this review of the scientific literature, we will focus on the role of quantitative movement analysis in neurodegenerative disorders and restrict our discussion to the measurement of gait and postural control measured during standing in key conditions. The review has four sections, each of which addresses a key aim. Section I aims to provide an overview of the strengths and limitations of current measurement techniques/devices, outcomes, and protocols relevant to key measurement needs. Section II aims to provide evidence to support the use of gait and postural control as clinical biomarkers as defined above, drawing from studies in Parkinson’s disease (PD), ataxia, and dementia. Section III aims to highlight new and emerging areas relating to bioinformatics (data mining and machine learning) and what we can learn in the context of disease classification, phenotyping, and risk prediction. Finally, Section IV offers recommendations for future work in this field. 

## 2. Section I: Quantitative Movement Analysis: From Measurement Tools to Outcome Measures

**Figure 1:**
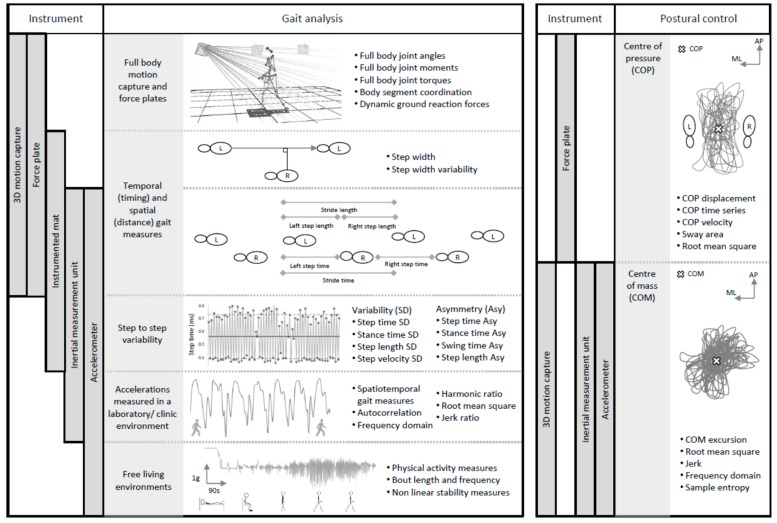
Summary of outcome measures that may be obtained from a quantitative assessment of gait and postural control.

Below, we provide a brief overview of the measurement tools, protocols, and quantitative outcome measures that are currently utilised, highlighting the most relevant (Figure 1 and Table 1).

### 2.1. Quantifying Gait and Postural Control—Which Tools?

For an objective, quantitative analysis of gait and postural control, three-dimensional motion analysis, footswitches, instrumented walkways, body-worn sensors, pressure mats, force platforms, posturography, and electromyography are the most common tools, each with their own strengths and limitations. Quantitative tools can be as simple as a stopwatch, which with a known distance, can be used to gain clinically relevant measures such as gait speed [5]. The main consideration when deciding the best approach is the need to balance the requirement for better granularity, sensitivity, specificity, measurement accuracy, and minimal rater bias, with the complexity and feasibility of using such methods in clinics, communities, and clinical trials (Table 1).

Three-dimensional motion analysis systems are capable of measuring movements of the whole body; they can measure both gait and postural control, are highly accurate and precise, and are relied upon as the gold standard to compare new tools as well as to evaluate the benefit of therapeutic interventions (e.g., surgical procedures, pharmacological therapies, assistive devices, and exercise training programs) [6]. However, the high cost, long preparation time, and need for specialist staff to operate these systems are barriers to their wholesale adoption within routine clinical care [7]. Furthermore, even when clinically implemented, the choices regarding protocols such as different marker sets and biomechanical models, which are needed to quantify kinetics and kinematics, combined with the complexity of the outputs, can greatly influence the outcome and decisions based on the data collected [8]. This means that these systems are largely limited to research settings.

Instrumented mats provide reliable quantitative spatiotemporal gait characteristics of the feet at a lower cost relative to three-dimensional motion analysis, while also providing pressure information [9,10,11]. They can provide a number of gait outcomes ranging from information on pace to dynamic postural control [12]. If located in a dedicated space, the preparation time for the device and the participant is minimal. Additionally, most systems come packaged with accompanying software that is capable of generating automated reports, meaning that specialist support may not always be required. Limitations include: the devices are not easily portable and require large indoor spaces, are not capable of measuring standing balance, and that due to the finite size of the mat, multiple trials are required to generate reliable gait measures [13]. 

More recently, technological advances in wearable devices allow an alternative to traditional laboratory-based and clinical assessments of gait and postural control. Wearable sensors are mobile devices that are designed to be worn on the body, or embedded into watches, bracelets, and clothing [14]. They may be used in the clinic, but also in free living. This opens a whole new perspective in terms of assessing mobility over extended periods of time [15,16,17,18] while concurrently evaluating traditional and novel measures of gait [19,20], quantitative measures of physical activity [21], and postural outcomes such as transitions and turns [22,23]. The inclusion of free-living data alone or in addition to clinical assessment provides vital information that may help inform the diagnosis and monitoring of neurological disease. However, whilst these new avenues and variables appear promising, the best combination of methods and metrics are still to be determined [15,24]. However, limitations with wearable devices should be considered. Although a single accelerometer is inexpensive relative to the other more traditional devices highlighted above, multiple sensor systems with accompanying software that is capable of calculating outcome measures into reports dramatically increase the price. Also, with such systems, clinicians require methodological training, which is critical for avoiding blind interpretations of erroneous reports. Lastly, although many proxy measures of gait have been validated [25,26], many algorithms for their estimate and novel measures have not, meaning that further effort is needed in order to create robust, validated, and population-specific normative values. 

### 2.2. Outcome Measures and Data Collection Protocols

Below, we provide a brief summary of some of the most common outcomes from quantitative analysis that are used to describe gait and postural control (Figure 1). Gait and postural control are intimately related. Gait models include measures that reflect aspects of gait-related postural control, and can be interpreted as such. However, for the purposes of this review and to reflect how assessment is commonly conducted, we address each independently.

For gait, the most common outcome is gait speed because of its robust clinimetric properties [5,12]. Often regarded as a global measure of overall function, gait speed is informative; however, it does not reveal the specific gait deficit (i.e., temporal or spatial control of gait), and as such is limited [12]. Moreover, in laboratory and clinical settings, gait speed may also be susceptible to the ‘Hawthorne effect’, whereby participants perform particularly well in controlled environments whilst being observed [27]. Many other outcomes can be used to describe gait, and may add greater specificity regarding differentiating between neurological conditions and increased sensitivity when evaluating subtle within-person changes [12,28]. To aid interpretation, normative values have been published and serve as reference data [29,30], and conceptual models of gait have been developed to provide a more structured approach [12,31,32]. 

The measurement of gait can be broadly grouped within a structure that captures: (1) spatiotemporal features that reflect a typical gait cycle, which is expressed as the average of multiple steps over a specified distance; and (2) dynamic features of gait, which represent the step-by-step inconsistency of spatiotemporal measures across these steps [12]. These dynamic features are typically represented by the within-person standard deviation (SD) or the coefficient of variation [33]. Hausdorff (2009) [34] also introduced a broader definition of the dynamic features of gait that incorporated the underlying structure and pattern of movements in gait derived from data collected over longer periods. These long-term, fractal-like correlations break down with age and disease, and as such, different measures reflecting the variability of movement may provide additional sensitive markers of early/prodromal disease [34]. 

Standardised protocols for the assessment of gait (for a recent review, see [35]) typically measure gait at a preferred walking speed over four metres, and are clinically applicable [36]. Departure from this protocol is acceptable for some gait characteristics, but not for others. Gait speed is reliable over 10 metres or six minutes, for both preferred and fast walking, and in clinic and home environments [26,37,38,39,40]. This is not the case for gait variability, where clinimetrics improve with a greater number of steps, and a minimum of 30 steps is advocated [13,41,42]. Walking over longer durations (e.g., two and six-minute walking tests) are also commonly used to infer endurance [43]. Protocols are also modified to provide additional challenge; these are so-called “stress tests”. They include dual-task paradigms, turning, backward walking, and walking at a fast pace [44]. Dual-task testing paradigms (i.e., asking someone to recall a sequence of numbers while walking) are employed to reduce the compensatory cognitive control of gait, revealing latent motor deficits; as a consequence, they also expose the level of compensatory cognitive control that is required to maintain gait performance. 

A range of outcomes exist for measuring postural control during standing. Typically, they are derived from either movements of the center of mass (COM) or the center of pressure (COP), and can be summarised as linear and non-linear outcomes (see Figure 1). Linear parameters and derived indexes provide information about the direction (e.g., anterior–posterior or mediolateral directions) and global ‘magnitude’ of postural sway (e.g., root mean square (RMS), limits of stability, jerk, ellipsis), and the fluctuation of COM or COP displacement (frequency domain metrics) [45,46,47,48,49,50,51,52]. Non-linear outcomes describe the regularity or predictability of balance control [53]. 

The protocols that are used to assess postural control vary greatly from quiet standing to standing barefoot or with shoes on, on a foam support or firm surface, with eyes open or closed (Romberg test), with either standardised or unrestricted foot placement, with arms across the chest or by their sides, and over different trial durations ranging from 30 to 120 seconds [48,51,53,54,55,56]. To date, postural control outcomes are typically summarised over the test duration, which may limit comparability across protocols of varied duration, as most linear metrics are time-dependent and thus influenced by test duration. For example, a person’s total COM excursion will increase relative to time, highlighting the need for normalisation and standardised protocols for between-investigation comparisons (for a detailed description of how method can impact postural control measures, please see [57]). Alterations in postural control over discrete windows of time may provide a more subtle reflection of postural adaptations in addition to outcomes averaged across the test duration [56]. 

In summary, the breadth of tools that are used to measure gait and postural control are vast, as are the range of protocols for data collection and the outcome measures that are extracted. Therefore, there is a need to standardise and harmonise approaches. Currently, the optimal testing battery for gait and postural control applied either independently or in combination is unknown, and further work is needed to define this on a disease-by-disease basis, and also with respect to the purpose (e.g., diagnosis, progression, risk prediction), as one size will not fit all. The opportunity for the uncontrolled continuous monitoring of gait and postural control during free-living activities is an area of considerable interest, and its additional measurement holds promise for the future. A growing number of outcomes may be obtained ranging from the micro (i.e., step length and time) to the macro (i.e., total time walking per day) features of gait. However, the optimal approach to integrate free-living movements into clinical decision making is yet to be defined, and this continues to be an area of emerging interest. Despite this, the promise of movement analysis for diagnosing and monitoring neurological conditions is becoming increasingly evident, and examples from recent literature are highlighted in Section II across a range of different disease groups. 

## 3. Section II: Distinguishing Features of Gait and Postural Control across Neurodegenerative Conditions

In this section, we highlight examples from the recent literature to illustrate the value of instrumented assessment of gait and postural control across a range of different disease groups. 

### 3.1. Parkinson’s Disease 

Parkinson’s disease (PD) is the second most common neurodegenerative disease after Alzheimer’s disease (AD), affecting one in every 500 adults in the United Kingdom (UK) and up to 10 million worldwide [58,59,60]. PD was previously described as a degeneration of dopaminergic cells in the substantia nigra; however, a more contemporary understanding of PD highlights that it is a complex multi-system disorder that is represented clinically by a syndrome with multiple neurotransmitter deficits (for a recent review, see [61]). A variety of clinical assessment scales have been designed to evaluate the motor symptoms of PD (including gait and postural control) such as the Unified Parkinson’s disease rating scale Part 3 (UPDRSIII [62]) and the Hoehn and Yahr scale [63]. They are embedded within routine clinical evaluation and categorically grade disease severity and motor symptoms from normal to severe. As such, they often do not capture detailed information regarding motor deficits, may miss subtle within-person changes, and can be susceptible to variation when administered by different assessors.

The quantification of gait characteristics in PD can inform risk [64], progression (including response to treatment) [65], and diagnosis [66]. Notably, discrete gait changes predict both future falls [67,68] and cognitive decline in incident PD [69], raising the possibility of a target for a preventative approach in early disease. Subtle and discrete differences have been identified in early PD compared to age-matched controls, with reduced step length, increased asymmetry, and step-to-step variability [65]. Gait impairments evolve over time from a subtle, discrete picture to a more global presentation of deficit in all its characteristics [65,70,71]. Subtle gait impairments are also present in individuals with Parkinson’s ‘at risk’ syndromes such as rapid eye movement (REM) and sleep behavior disorder (RBD) prior to the development of Parkinsonian features (so-called ‘prodromal’ disease) when compared to healthy controls without risk factors [72]. 

Monitoring upper-body movements (such as the magnitude of arm swing or movement of the trunk) during walking is emerging as a powerful measure complementary to traditional gait analysis (measuring stepping characteristics), and has been shown to be capable of discriminating PD from controls [24,73], and PD fallers from non-fallers [74,75,76]. Arm swing variability during gait has also been identified as a distinguishing feature in carriers of the G2019S mutation, which was significantly different to both non-carriers and people with PD [77]. This has raised the possibility of upper body movements during gait as a clinical biomarker for PD to enhance diagnostic accuracy, which in early disease may only be between 70–80% [78], and supports the use of quantitative, objective assessments to measure changes that may not be detected during routine clinical observation. 

Gait continues to deteriorate even in the early stages of the disease despite optimal medication with evidence to suggest that some discrete characteristics of gait are dopa-resistant (i.e., step length, step width, and swing time) [65,71]. Other gait characteristics appear to be responsive to intervention, with a recent review of pharmacological therapies highlighting the role of cholinesterase inhibitors to improve gait variability [79]. Therefore, quantitative movement analysis may be useful when understanding the effectiveness of levodopa, and potentially highlight when alternative treatment options may be required. Deficits in gait have been linked to primary pathophysiology, as visualised with functional and structural neuroimaging, cerebral spinal fluid, and blood-based biomarkers, supporting the use of discrete gait characteristics as potential clinical biomarkers to track pathology. Gait impairments such as gait speed may be attributed to underlying cholinergic dysfunction [80,81], with evidence to suggest that amyloid proteinopathies may also contribute to the progression of dopa-resistant gait characteristics (step time and length variability) [71]. Freezing of gait (FOG) is a debilitating symptom that often affects patients with advanced PD, and has shown a positive response to levodopa [67]. FOG is recognised as an episodic absence or marked reduction of forward progression of the feet, despite the intention to walk [82], and is associated with an impaired regulation of stride variability [83,84,85,86]. A recent report explored the potential use of smartphones to assess digital biomarkers of PD, including gait and postural control, and to identify exploratory outcome measures for Phase I clinical trials [87]. The results revealed acceptable adherence and moderate to strong retest reliability (Intraclass Correlation Coefficient = 0.84), highlighting the potential of using smartphones to collect gait and postural control data. Not only were people with PD distinguished successfully from controls, the analysis of turning (possible with accelerometer and gyroscope-derived measures) offered increased sensitivity compared to traditional clinical scales [87].

Previous research quantifying the linear parameters of postural control has suggested that: (i) abnormalities in postural control during quiet standing exist even in early PD [45,46,56]; (ii) linear parameters can differentiate between PD motor subtypes [88]; and (iii) as disease symptoms progress, sway parameters deteriorate, especially in the mediolateral direction [45]. The positive effect of dopaminergic replacement therapy that has been observed for gait may not be paralleled for postural control, where levodopa has been shown to worsen some outcomes [50]. Non-linear measures of postural control have shown that people with PD display lower predictability/regularity of the COM along all sway directions; this may be explained by the loss of constant fine adjustments of posture due to impaired sensorimotor integration and the disturbance of habitual motor control pathways [53]. However, it is still unclear whether regularity metrics are sensitive to disease progression [53,89]. However, practically, monitoring the positive and negative influence of therapies may be useful in clinical management and falls risk.

### 3.2. Ataxia 

The prevalence of hereditary cerebellar ataxias is estimated at 2.7/100,000 (average derived from meta-analyses [90]). Ataxia describes a collection of neurological disorders affecting the cerebellum that impair the control and coordination of whole body movements, eye movements (nystagmus), and speech (dysarthria) [44,91,92]. Consequently, the integration of sensory information to coordinate voluntary movements is challenged, and impairs gait and postural control. Poor gait control is often the initial symptom in ataxia groups [93], reportedly occurring in around 60% of ataxia patients [94], and in some cases emerging prior to the onset of neurological symptoms [95]. Quantitative movement analysis has shown that ataxic gait is associated with slower walking speeds and a reduced cadence, a shorter step and swing phase duration, a longer double limb support phase, wider steps, and increased gait variability (particularly step length and width) [96,97,98,99,100,101,102,103,104]. Impaired gait and postural control are associated with an increased falls risk [99,105,106], and serve as attractive targets for intervention (refer to [107,108] for comprehensive reviews of gait and balance, respectively). Falls are common in people with ataxia, occurring in up to 74%, and prevalence is proportionate to other neurological conditions such as PD [109]. We draw upon examples of inherited and secondary ataxias [110,111] to highlight the importance of quantitative analysis of gait and postural control in this patient group. While broad clinical scales such as the Scale for the Assessment and Rating of Ataxia (SARA) [112] provide an indication of overall function, they are unable to reveal the nature of subtle movement impairments in this patient group. Quantifiable, objective measures that may be used as markers to document gait and postural impairment are lacking when relying on these clinical scales alone. Gait variability, in particular variability in the timing of movement, is specific to cerebellar dysfunction [113], and cannot be assessed using clinical scales. 

Patients with ataxic symptoms, including individuals with multiple sclerosis, display deficits in postural control including a greater magnitude and speed of postural sway [97,114], which is attributed to a reduced range of motion at the knee (‘locked knees’) and a delayed response in muscle activity [115]. To compensate for this poor postural control, people with ataxia often widen their stance (base of support) to stabilise the head and trunk [116]. Anterior–posterior falls are more common than falls in the mediolateral direction [117]. Accordingly, assessment protocols that assess balance in the anterior–posterior direction pose a heightened challenge for people with ataxia [118,119]. 

Quantifiable measurement outcomes obtained during gait are useful for distinguishing ataxia and mitochondrial disease from controls and other neurological conditions such as PD and hereditary spastic paraplegia (HSP) [100,120,121]. For example, patients with mitochondrial disease walk slower, with a shorter step and increased step width variability during normal and dual task walking compared to controls [120]. In contrast, patients with ataxia walk with an increased step width and larger ankle range of motion compared to controls, PD, and HSP [100]. Quantifying gait using wearable sensors is a valid measure for use with mild to moderate ataxia [26], with the mediolateral acceleration of the upper body during gait in particular being specific to disease and sensitive to symptom severity. Therefore, this may serve as a clinical biomarker for ataxia [122]. Gait outcomes extracted using more complex non-linear and data-driven approaches also offer potential, and are able to differentiate ataxias from other neurological patient groups [99,100,103]. Complex walking tasks such as incline walking [123], obstacle avoidance [124,125], and turning [102,122,126] may serve as ‘stress tests’ to exacerbate underlying gait impairments in ataxia and identify preclinical changes in this patient group [127].

### 3.3. Dementia 

Dementia is a neurodegenerative syndrome that is characterised by multiple cognitive impairments affecting social and/or occupational functioning [128,129]. Globally, almost 50 million people are affected, the majority of whom are aged over 65. Risk increases exponentially with age, leading to high socioeconomic costs [130,131]. Dementia has many subtypes driven by different pathologies, the most common incorporating: Alzheimer’s disease (AD); Lewy body dementias (LBD), which is comprised of dementia with Lewy bodies (DLB) and Parkinson’s disease dementia (PDD); and vascular dementia (VaD). Mild cognitive impairment (MCI) may also be a risk factor for dementia, and can also be classified into subtypes [129,132]. The early and accurate identification of dementia and its subtype is of importance; however, it remains clinically challenging. Improved differentiation is critical, as misdiagnosis can lead to incorrect treatment and management of disease [133]. This is particularly pronounced between LBD and AD due to shared clinical features and cross-pathology [134,135,136]. DLB is pathologically classified by the presence of Lewy bodies containing abnormally folded alpha-synuclein within the brain. Clinically, it is differentiated from AD by prominent deficits in attention, visuospatial and executive function, cognitive fluctuations, visual hallucinations, RBD, and parkinsonism [133]. 

Gait, rather than being an autonomous task, is under cognitive control due to shared neural networks, and is evident in dementia cohorts [137]. Slow gait precedes and predicts cognitive decline and dementia, with gait impairments occurring up to nine years prior to diagnosis [138]. Even in the early stages of cognitive impairment, people with MCI walk slower, with shorter steps and increased variability compared to cognitively intact older adults [139,140]. People with dementia walk slower, with shorter strides and increased stride time variability compared to controls, and this increases with disease severity [140,141,142,143,144]. A recent review of gait across common dementia subtypes revealed slower gait and impaired timing (i.e., longer stance, stride, double support time) in people with AD, LBD, and VaD compared to controls [145,146,147,148], and demonstrated some evidence for a greater variability of gait in people with AD [149,150,151,152,153]. Falls risk is also increased in dementia and MCI, with DLB and PDD subtypes reporting the greatest risk [154]. An important link has been demonstrated in those with preclinical AD and falls, highlighting a possible underlying pathological basis, as amyloid burden predicted falls risk [155]. Discrete gait characteristics may serve as a useful tool for distinguishing between dementia subtypes [31], and can thus aid diagnosis. Differences between subtypes include slower gait in VaD compared to AD and slower pace, impaired timing, and an increased variability of gait in LBD compared to AD [145]. There is evidence to suggest that gait impairments differ across MCI subtypes [153], supporting a role for discrete gait outcomes as clinical biomarkers to aid diagnosis. 

Only a small number of studies have looked at postural control in older adults with cognitive impairment, making it difficult to draw robust conclusions. A recent review reported impaired postural control in MCI [139] and AD [156]. However, group differences are not consistent [156]. More research may demonstrate postural control assessment, in addition to gait analysis, as a useful biomarker of cognitive impairment. 

More recently, the monitoring of gait using body-worn sensors as in other neurological diseases has been explored in people with dementia. A recent study showed that clinic and home-based monitoring was feasible and acceptable in dementia populations, and trends suggest that gait impairments such as greater variability and a slower pace can differentiate dementia from controls when measured in free-living environments [157,158]. This shows potential use for monitoring gait prior to dementia onset and throughout the progression of the disease, providing valuable insight into the utility of gait as a clinical tool for the diagnosis and monitoring of dementia.

### 3.4. Summary

The current literature suggests that measuring gait and postural control has utility as a clinical tool, both for supporting diagnosis and monitoring disease progression. Impairments in gait and postural control may be the first manifestation of underlying neurological disease, such as in Parkinson’s disease, ataxia, and dementia. There is evidence for the role of gait analysis in predicting and identifying the onset of cognitive decline in Parkinson’s disease, and emerging evidence for the use of gait as a possible biomarker of dementia subtype. The early accurate diagnosis of these neurodegenerative conditions is a key target within clinical research, and the recent emergence of inexpensive wearable technology for analysing gait and posture has potential to be deployed as a widespread diagnostic tool. Quantifying gait and postural deficits can also be informative towards fall risk, which is a common problem in neurological conditions. Gait and postural measurements are increasingly used for disease progression monitoring, and may form an important part of a digital endpoint in clinical trials. Wearable technology and quantitative clinical measures may lead to improvements in the accurate identification of diagnosis, and may highlight individuals who would benefit from targeted intervention. Lastly, there is the opportunity to combine movement-based measures with biochemical and genetic analysis, such as in PD, where carriers of the autosomal dominant G2019S showed significant changes in gait variability compared to non-carriers [159].

## 4. Section III: Emerging Techniques for Disease Classification and Risk Prediction—So-Called ‘Big Data’ Approaches

**Figure 2:**
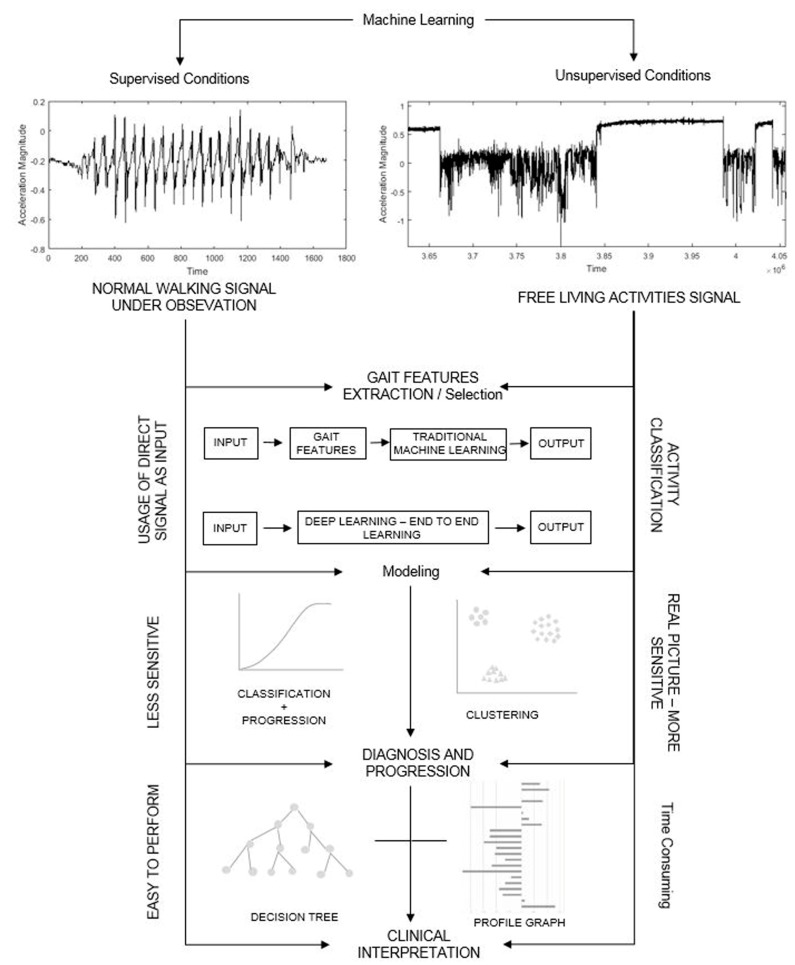
A machine learning end-to-end framework for the analysis of gait dynamics in the laboratory and community.

It is evident that there is a plethora of measurement outcomes that are used to describe gait and postural control that are often used generally across a range of neurological conditions. Typically, a univariate approach is adopted, whereby measurement outcomes are considered independently, which may increase the risk of losing important information. Developing methods to reduce the number of (gait or postural) measurement outcomes included within statistical models is needed. Data-driven approaches that apply machine learning principles are beginning to explore the optimal combination of characteristics that successfully classify patients by condition (Figure 2) to improve diagnostic accuracy [160]. Recent work has used gait characteristics for fall classification in people with PD [161], and sensitivity analysis for feature selection when classifying PD [162]. A variety of data mining and machine learning approaches have been used to classify neurological conditions using gait and postural control data. For example, support vector machine techniques identified patients in the early stages of PD using their step length, which was measured during gait [163]. Multiple layer perception neural networks have distinguished Friedrich’s ataxia from controls using stride time and gyroscope-derived outcomes [164]. Postural control measures have demonstrated utility for distinguishing AD from controls [165]. Adopting these analytical approaches also allow for gait and postural control outcomes to be considered in combination rather than independently. However, further research is required to select the appropriate gait and postural control characteristics for each disease type. This will aid clinical interpretation, reduce computational demand, and improve classification accuracy [12,166]. 

Realistically, in the future, wearable sensors will be the most practical tool to use to capture gait and postural control. However, gait and postural control data derived from wearable sensors is complex, multidimensional, and has high patient variability (no two patients are alike). Therefore, there is a need to find measures that offer increased sensitivity for distinguishing between neurological conditions at each disease severity level while controlling for between-person variability. Promising attempts to model and classify dementia and PD using measures of gait and postural control with a variety of classification tools (e.g., support vector machines, hidden Markov models, multilayer layer perception, neural networks, etc.) have been reported [163,164,165,167,168,169,170,171]. Even though the perfect classification accuracy is reported with various techniques, the optimal method or combination of approaches has not been identified, much less tested. In addition, robust modelling has not been possible, because studies are often limited to data collected in small, poorly described patient cohorts. Efficient systems for computational processing, the visualisation of multivariate gait and postural control profiles, and disease modelling in a clinician-friendly format are also essential for clinical adoption. There are currently no established tools for identifying and detecting disease or modelling disease progression in neurological diseases such as those described in this review. This is currently an area of significant interest.

## 5. Section IV: Recommendations and Future Direction 

A growing interest and body of literature is evident in the area of gait and postural control measurement. Complex techniques such as motion capture are unlikely to be deployed in clinical settings on a large scale in their present form due to their considerable cost, the dedicated personnel/expertise required, and potentially lengthy data collection and analysis period. However, techniques that reduce participant preparation time are currently being developed (i.e., markerless motion capture [172,173,174]) and aim to drive down financial cost, improve accessibility, reduce data collection time, and ultimately increase productivity. Although showing promise, similar to wearable sensors, research into the accuracy and validity of each device is paramount before integration in routine clinical practice. Furthermore, continued research should strive towards continued algorithm development to provide the most robust sensitive measures to clinicians so as to overcome the current pitfalls of the technology. The ultimate goal of community-based methods to quantify gait and postural control is to characterise clinical populations on a global scale and revolutionise current healthcare practises. Remote monitoring offers the opportunity to put the patient at the forefront of his or her own healthcare and management, and provide timely and effective intervention. First, to achieve such progress, it is imperative to ensure that the platform (device, outcomes, protocols, analytical pipelines, and processes) for collecting this information is robust, and personal data remains secure. This will allow the creation of normative databases, increasing prognostic capacity and providing a comprehensive understanding of the clinical landscape and therapeutic needs. Novel therapeutic interventions are required that are personalised, targeted to specific gait and postural impairments, and ultimately effective.

Future challenges include disentangling the process of ageing from the accelerated process of neurodegeneration whilst accounting for individual variation, comorbidities, lifestyle, overlapping sequelae, and atypical disorders, etc. To achieve this goal, the investigation of deep/machine learning techniques that have the potential to include other non-movement analysis-derived biomarkers appears to be a worthy pursuit. As such, more epidemiological studies are required to understand the interaction between lifestyle factors, individual capacity, and the environment [175] to improve prognostic and diagnostic accuracy. Collecting and aligning prodromal and disease cohort studies through dedicated consortia will enrich our current understanding of biomarkers and risk factors. Incorporating post mortem data retrospectively would be beneficial to verify the underlying pathology in complex conditions.

### Recommendations

- Quantitative, objective assessments of gait and postural control should supplement traditional disease-specific scales in clinical trials to aid diagnostic accuracy and patient monitoring.
- Education around the advantages and disadvantages of quantitative analysis should be available to allow the clinician and clinical academic to make an informed decision about the best tool, protocol, and outcomes.
- Continued efforts are needed to validate the optimal protocol and outcome measures to best inform clinical management and research, and this requires a discrete condition-based approach.
- Further research using machine/deep learning should be explored to advance opportunities for optimised diagnosis and disease monitoring.
- Development of normative values across a range of standardised outcomes will help interpret gait and postural control outcome measures, and embed their clinical use and a personalised approach to management.
- Further research is needed in order to validate gait and postural control as approved disease biomarkers and progression markers.

## Figures and Tables

**Table 1:** A summary of the advantages and disadvantages of measurement devices used to quantify gait and postural control. COP: center of pressure.

Device	Advantages	Disadvantages
3D motion capture	- Considered the gold standard - Highly precise and accurate - Potential to measure a large variety of outcomes - Non-invasive - High-resolution data	- High cost - Requires experienced technical expertise - Requires a large purpose-built dedicated space usually limited to laboratory/research environments - Participant preparation can be time-consuming
Force plates	- Considered gold standard for measuring ground reaction forces and COP - Non-invasive - Minimal space required - Minimal participant preparation time - High-resolution data	- High cost - Requires experienced technical expertise - Requires a purpose-built dedicated space
Instrumented mats	- Minimal processing time - Non-invasive - Minimal participant preparation time - Portable	- Extractable features are limited by mat dimensions - Requires a large space to accommodate the mat dimensions - Limited to temporal spatial and foot pressure gait outcomes of the lower extremities
Inertial measurement units	- Capable of capturing continuous movements in laboratory and community environments - Non-invasive with minimal participant preparation time - Certain systems provide automated reports - Cheaper than the gold standard - Portable	- Often requires complex algorithms and special expertise to extract key features - Features are often indirect measures requiring additional participant measurements - Free-living measurements may be limited by recording time (if battery powered) or data storage (if data is stored internally on the device)
Accelerometer	- Low cost - Wearable, wireless technology capable of capturing continuous movements in lab and community environments for prolonged periods (>one week) - Non-invasive - Minimal participant preparation time - Portable	- Often requires complex algorithms and special expertise to extract key features post-data collection - Features are often indirect measures requiring additional participant measurements - Data collected in community living environments lack context
